# Palpation of the Respiratory System in Osteopathic Manual Medicine: From the Trachea to the Lungs

**DOI:** 10.7759/cureus.18059

**Published:** 2021-09-17

**Authors:** Bruno Bordoni, Allan R Escher

**Affiliations:** 1 Physical Medicine and Rehabilitation, Foundation Don Carlo Gnocchi, Milan, ITA; 2 Anesthesiology/Pain Medicine, H. Lee Moffitt Cancer Center and Research Institute, Tampa, USA

**Keywords:** fascintegrity, lungs, diaphragm, fascia, visceral osteopathy, osteopathic

## Abstract

There is a lack of published literature in osteopathic manual medicine on how to perform palpation of the lower respiratory tree such as the trachea, main bronchi, and lungs. Several authors have studied the osteopathic effect and respiratory response of palpation but have failed to demonstrate how to perform palpation of the visceral areas involved in breathing, either in the context of a clinical trial or as a case report. This paper reviews the innervation of these anatomical areas, the mechano-metabolic weight of the passage of fluids and air in the respiratory tract, the anatomical topography, and the movements involved in respiration. Drawing from current knowledge, this article illustrates, for the first time, how to place the hands for an effective osteopathic assessment of the tracheal, bronchial, and pulmonary structures. Understanding how to perform palpation of the lower areas is a fundamental tool in the clinic and potential therapy in osteopathic manual medicine.

## Introduction and background

The visceral component of osteopathic manual medicine (OMM) has received scientific interest from the second half of the last century [1-3]. Among the visceral OMM pioneers from the first half of the twentieth century is Louisa Burns, who conducted experimental research on visceral and somatic reflexes [4]. To our knowledge, there has been no reference in the literature to palpation of the pulmonary system, trachea, and bronchi in the context of OMM. It is unthinkable to plan osteopathic treatment without an evaluation prior to and following manual work performed by a clinical osteopath. Osteopathic treatment can improve well-being and reduce respiratory symptoms, without necessarily improving all parameters measured by instrumental evaluations [5-7]. This means that manual evaluation does not simply take machine-readable data into account; very often, tactile data allow the movement of one motor area to be compared with that of another. Assessing the ability of different tissues to move under the hand is essential for understanding how and with which technique to increase the fascial space using an OMM approach [8]. Increasing the space of movement between different layers of tissues makes it possible to manipulate the local metabolic framework and the activity of afferent pathways, which can change a patient’s clinical and/or symptomatic status [8]. Space allows for more movement, and movement allows for adaptation to one’s environment. Adaptation is necessary for life. The main goal of osteopathy and manual therapy is to create space between different tissues. The sliding capacity of the various tissue layers and between the different body components, which allows for movement between cells, is considered as a salutogenic factor that facilitates the circulation of fluids, biochemical exchanges, and the adequate management of multiple internal and external stimuli that can disturb the body [8]. To our knowledge, no previous studies on the OMM approach have described the manual evaluation on respiratory tissues in any detail and have instead focused on structural and/or instrumental examination [7,9-12]. For example, the forced expiratory volume in 1 second (FEV1), which is a respiratory value obtained from spirometry, is not necessarily related to the presence of dyspnea, physical performance of patients with chronic obstructive pulmonary disease, or to the functionality of the respiratory muscles [13-14]. The manual treatment of the thoracic outlet for thoracic outlet syndrome has been reported to improve respiratory parameters [15]. Furthermore, it is important to remember that dyspnea and breathlessness are not always related to a real lung problem but can derive from psychological dysfunction or from a lesion of the central nervous system, which creates a vicious cycle that negatively influences respiratory function and interoceptive neurocognitive mechanisms [16-17]. Knowing how to properly perform palpation of the respiratory structures can allow osteopaths to determine whether there is a real dysfunction of the respiratory tract and provide fundamental insights that can inform clinical interpretations. The present article reviews the anatomical and neurological knowledge of the respiratory system, from the trachea to the lungs and their topographical anatomy, with the aim of defining a palpation OMM approach that is in line with the current literature and fills the gap in osteopathy literature.

## Review

Trachea, bronchi, and lung innervation

The trachea and bronchi are part of the lower respiratory tract and are richly innervated by efferent and afferent tracts; the efferent pathways originate from the parasympathetic system while the afferent pathways originate from neural cells found in the dorsal root ganglia (T1-T6) and/or in the ganglia of the vagus nerve (jugular and nodular ganglion) [18-19]. Vagal efferents travel to ganglia within the trachea and bronchi to regulate smooth muscle and mucus glands [20]. Through cholinergic signaling (acetylcholine), the vagus nerve activates smooth muscle (the bronchoconstrictor effect) and stimulates vasodilation and mucous secretion [18]. The right and left vagus nerves run through the cervical area within the carotid fascia (together with the internal jugular vein and internal carotid artery); before entering the mediastinum, the right nerve passes the right subclavian artery (in the posterior proximity of the sternoclavicular joint area and at the level of T1-T2) and creates a recurrent laryngeal accessory branch known as the inferior laryngeal nerve, which reaches the tracheoesophageal groove, slightly anterior to the inferior laryngeal nerve, and is enveloped by the tracheoesophageal fascia [21-22]. The left recurrent laryngeal nerve originates within the mediastinum, passing the ligamentum arteriosum or arterial ligament (between the aortic arch and pulmonary artery) to the tracheoesophageal groove, slightly anterior to the pulmonary artery, and is enveloped by the tracheoesophageal fascia [21-22]. The inferior laryngeal nerve of the right and left vague nerves contains fibers that originate from the XI or accessory cranial nerve [23]. To a lesser extent, sympathetic fibers from the stellate ganglion and superior cervical ganglion also reach the trachea and bronchi and, through noradrenergic signals and neuropeptide Y, can influence the submucosal glandular response and arterial ducts [18-19]. The afferent pathways innervating the trachea and bronchi are more complex. Afferent pathways comprise unmyelinated vagal type C fibers (which send pain signals), partially myelinated fibers that send mechanical information (A-delta type), fully myelinated (A-beta type) fibers, and fine nerve fibers that form a network or plexus [24-25]. Fine nerve fibers are further divided into intraepithelial fibers and subepithelial fibers, which correspond to fibers that innervate the mucosa and those that innervate smooth muscle, respectively [25]. There are also vagal non-adrenergic, non-cholinergic-type fibers that can relax smooth muscle; these originate from the myenteric plexus of the esophagus (particularly from the trachea) [26]. The epithelium of the trachea and bronchi contain neural bodies or neuroendocrine lung cells in the vicinity of the afferent pathways that influence parasympathetic pathways in an immunological, central, and peripheral context [18,27]. The broncho-tracheal afferents send mechanical and nociceptive information, such as the stretching that these structures undergo during inhalation and exhalation or in the presence of inflammation, respectively, to the central nervous system. Vagal afferents arriving at the nodose ganglion stimulate neurons of the nucleus tractus solitarius and then of the nucleus ambiguus while the afferents arriving at the jugular ganglion stimulate neurons of the spinal trigeminal nucleus [20,28]. Sympathetic fibers are sent from the locus coeruleus to correctly manage the vagal information of the vagal nuclei located in the brainstem (nucleus tractus solitarius and ambiguus) [20]. Parasympathetic afferents can transmit information about non-physiological stimuli, such as pain or the presence of irritants and cough if there are mechanical forces that alter the mechano-metabolic environment of the entire tracheobronchial tree due to local and systemic disorders [28]. Non-physiological mechanical stimuli can alter the vagal tone and efferent responses, which compromise a functional respiratory rhythm and cause various symptoms that are apparently unrelated to the airways such as a decline in mood or a low pain threshold [18-19,28-30]. Palpation and treating the airways using an osteopathic approach is useful not only for dealing with respiratory problems but also for managing both local and more complex symptoms. Concerning the innervation of the pleura, the parietal pleura differs from the visceral pleura, in that the former receives somatic innervation with a higher mechanical sensitivity [31]. The thoracic spinal nerves innervate the parietal pleura of the costal, cervical, and mediastinal area while the diaphragmatic portion of the parietal pleura is innervated by phrenic nerves [31]. A disturbance of the costal parietal pleura can alter sensitivity (or pain) of the skin of the chest, and the pleural cervical area can be a source of problems related to the first thoracic nerve and the skin area of the arm; dysfunctions of the diaphragmatic pleura can increase skin sensitivity and pain and impair the joint movement of the chest and shoulder area [31]. The lower portion of the diaphragmatic pleura also receives innervation from spinal nerves of the T6 tract; the presence of altered parietal pleural afferents can cause disturbances in the abdominal area [31]. Scattered sympathetic and parasympathetic fibers can also be found in the diaphragm [32]. The visceral pleura has sensitive fibers in specific areas (peri-hilar, interlobar, diaphragmatic and mediastinal, and costal), which comprise parasympathetic pathways [33]. Each area innervated by vagal fibers has specific morphological and biochemical characteristics, a discussion of which is beyond the scope of this article [33]. The information collected by the vagal system, within the visceral pleura, is relayed by somatic and visceral afferents towards the centers that regulate breathing [33]. The neural involvement in speech is more complex, as the vagal endings can innervate ipsilateral or contralateral areas, or there may be a predominance of innervation; for example, the triangular ligaments (right and left) are primarily innervated by the right vagus nerve while the dorsal area receives few vagal fibers compared to the other portions of the speech complex [33]. More studies are needed to verify the effect of this neurological distribution in terms of symptomatology. The afferent pulmonary pathways, albeit with different modalities and different receptor types, send multiple sensory information to the same central pathways in which the tracheobronchial information is processed [34]. The central nervous system allows the afferent and efferent airways of the respiratory tract to meet. The intrapulmonary parasympathetic innervation originates from the ambiguus nucleus and in part from the dorsal motor nucleus [35]. Sympathetic innervation is from the intermediolateral and intercalated nuclei of the medullary thoracic area; the actions of these nuclei are influenced by brain areas such as the hypothalamus, reticular formation, and respiratory nuclei of the brainstem [35]. Sympathetic postganglionic pathways involving the lungs originate from the stellate ganglion and ganglia of the T2-T4 thoracic sympathetic chain [35]. Pulmonary sensitivity arises from innervation from A-delta fibers (myelinated fibers underlying mechanical sensitivity) and type C fibers (unmyelinated fibers underlying chemical, thermal, and, to a lesser extent, stretch sensitivity); the latter represent about 75% of the fibers present in the lungs [35-36]. Non-adrenergic non-cholinergic-type pathways can also be found in the lungs [36]. When the sympathetic and parasympathetic pathways enter the lung (at the level of the pulmonary hila), they form the pulmonary plexus [36]. The branches of the vagus nerve contain bronchoconstrictor and vasodilator fibers; those of the sympathetic pathway have bronchodilator and vasoconstrictor fibers. Vagal fibers release acetylcholine (ACh), which activates muscarinic receptors and smooth muscle contraction and increases the production of secretions by epithelial cells [35]. Vagal fibers can also release vasoactive intestinal peptide and nitric oxide, releasing smooth muscle (in association with the myenteric plexus) [35]. Type A vagal fibers are directed toward the mechanoreceptors of smooth muscle and within the airway epithelium. Type A fibers involve receptors such as ATP receptor P2X type 3, Na(+)/K(+) - ATPase alpha3, vesicular glutamate transporter 1 (VGLUT1), and VGLUT2 [35]. Furthermore, they may involve specific channels for mechanotransduction from mechanical stimuli, such as the two-pore domain K (+) channel TRAAK [35]. Type A fibers can be of the slow type (slowly adapting stretch receptors) and of faster type (rapidly adapting stretch receptors); these differences allow the vagal endings to act better for the management of pulmonary inflation [35]. There is a third type of type A fibers, referred to as "cough receptor," which involves extrapulmonary airways areas, and these fibers are activated by mechanical stimuli such as stretching [35]. Type C vagal fibers innervate type I interferon receptors (cough stimulation), and these fibers are stimulated by chemical factors; they are slow and unmyelinated fibers [35]. Type C fibers can synthesize some substances, such as tachykinin substance P and calcitonin gene-related peptide; the latter two substances can stimulate an inflammatory environment [35]. Type C fibers involve other types of receptors, such as receptor potential vanilloid 1, P2X2 (a purinenic receptor), receptor tyrosine kinases type A and B, receptor alpha-3 of the glial cell-derived neurotrophic factor family, and G-protein coupled receptor; we do not know in detail the functions of these receptors [35]. Post-ganglionic sympathetic fibers produce norepinephrine, which can produce enzymes such as neuropeptide Y and tyrosine hydroxylase [35]. The sympathetic function for the respiratory tract is to bronchodilate [35]. During inspiration, the vasodilatory action of the vagus and bronchodilator of the sympathetic pathway prevail; during exhalation, the vasoconstrictor of the sympathetic pathway and bronchoconstrictor of the vagus nerve prevail [36]. Neuroimmune regulation of the lungs is entrusted with great importance to vagal type C fibers through the release of acetylcholine or neuropeptides [37]. For a more in-depth description of the vagal receptor types present in the respiratory system, we recommend a recent article by Kupari and colleagues [38]. When we perform palpation of the respiratory tract, indirectly (from the skin), we can deal with the central and peripheral nervous systems.

Fluids and air flows

Air flows and fluid movements affect the shape and function of the respiratory tract. It is known that fluids manage morphogenetics during embryogenesis; in the formation of lung buds, their shape is determined by the pressures of various fluids, such as amniotic fluids and those secreted by the epithelial cells themselves [39]. In adult lungs, there are various fluids, such as blood, lymph, and pleural fluids. The pleurae are elastic tissues that contain different blood and lymphatic pathways; the visceral pleura has capillaries with a larger diameter and has a greater blood supply than the parietal pleura, with blood drainage performed by the pulmonary veins [40]. With respect to the visceral pleura, the parietal pleura has stomata (small openings) that collect the lymph fluid and send it into spaces such as lacunae; through lymphatic pathways that are more prevalent in the caudal areas, lacunae can send a large quantity of lymphatic fluid to the diaphragm muscle, passing through the triangular ligaments [40-42]. The fluid found between the two pleural sheets (maximum diameter 5-35 µm) originates from the capillary filtration of the parietal pleura into the interstitium, to eventually reach the pleural space; the stomata have the task of draining fluids (about 89%) from the pleural space [40,43]. The relationship between the triangular ligaments of the lung and the diaphragm muscle plays an important role in osteopathic management and evaluation. The pleural fluid or vascular microfiltrate is cleaned by the lymphatic pathways of the parietal pleura, and this mechanism occurs in the interstitium of the body organs [43]. The quantity of fluids and their distribution vary according to body position and respiratory and cardiac activities; in the prone position, the distribution is more homogeneous while in the supine position, the pressure of the lungs against the dorsal area and the weight of the heart push the fluids more laterally and in a craniocaudal direction [43]. The pressure of these fluids is greater at the apex of the lung [43]. The fluids allow the efficient transmission of mechanical information from the lung in all directions to the spine, simply by varying pleural fluid pressure [43-44]. The pressures generated by the fluids in the lung costal area are reduced during inspiration while pressure in the areas of apposition of the pleura (pleura-diaphragm-ribs) increases; the opposite happens with exhalation [43-44]. A difference in pressure has been linked to the pleural fluids, whereby the left lung shows slightly more negative values than the right; furthermore, a spike in vertebral pressure values has been reported at T7-T9 [44]. These pressures affect the position and movement of the thoracic spine (erection and rotation) [44]. Notably, all lung fluids are in contact with all body fluids, and vice versa, through a fluidic network that rapidly relays mechanical and metabolic information [45]. The difference in fluid pressures created by the movement is felt by the tissue, which adapts to defend its function/form; this process is part of the mechanism of mechanotransduction [46]. The movement of the fluids that influences the pressures of the tissues, including the respiratory tree, also influences the results of palpation via the quality of the perceived movement of the body areas. An excess or lack of in the quantity (and therefore movement of) fluids negatively affects the freedom of movement of the tissues. The passage of the inhaled air creates flows, which can affect the pressures felt by the tissues. Computational fluid dynamics studies have shown that the air entering the trachea (originating from the larynx) increases the flow rate during inhalation while decreasing the pressure of the same airflow; the result is the so-called jet stream in the trachea [47]. As the air descends into the trachea, the flow becomes more asymmetrical until it divides due to the deviation of the bronchi; a greater amount of air enters the right bronchus, despite the greater speed of the airflow at the entrance to the left bronchus [47-48]. The passage of the airflow and its pressure will determine the shape and function of the respiratory tree, as the mechanical stimulus perceived by cells that make up the respiratory tract (trachea-bronchi-lungs) will reflect the morphology and speed of the air itself [49-50]. The cyclic respiratory movement maintains the tone of smooth muscles, that is, the continuous passage of air guarantees a constant lengthening-shortening of the tracheobronchial tree with salutogenic stimuli [51]. If the dynamics of the airflow change, the shape and function of the respiratory tree change, leading to different pathologies [52-53]. If a clinician senses abnormalities of tissue movement during palpation, the cause may be related to changes in the shape and function of the respiratory shaft resulting from changes in airflow.

Topographic anatomy

**Figure 1:**
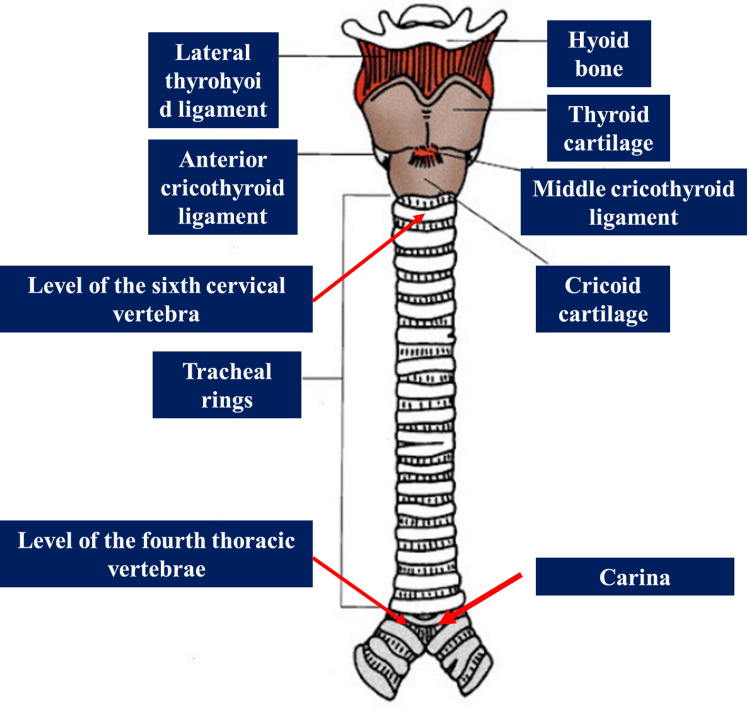
The figure illustrates the path of the trachea and some anatomical details. The figure highlights, in particular, the levels of the topographic tracheal anatomy. Property of Bordoni Bruno

The trachea begins after the cricoid cartilage (at the level of the sixth cervical vertebra) up to the carina at the level of the fourth thoracic vertebra (posteriorly), and at the level of the Louis angle (anteriorly); the trachea is approximately 15-10 cm in length [54-56]. The tracheal rings can be felt above the sternal notch for a maximum of 5 cm (the thyroid gland is higher up); inside the mediastinum, the trachea is slightly shifted to the right side, where it forms the carina and bifurcation of the bronchi [54]. The tracheal tract in the mediastinum is covered by the sternal manubrium [55]. With advancing age, the trachea tends to become less elastic, less mobile, and with calcification phenomena and morphological alterations (Figure 1) [56-57].

**Figure 2:**
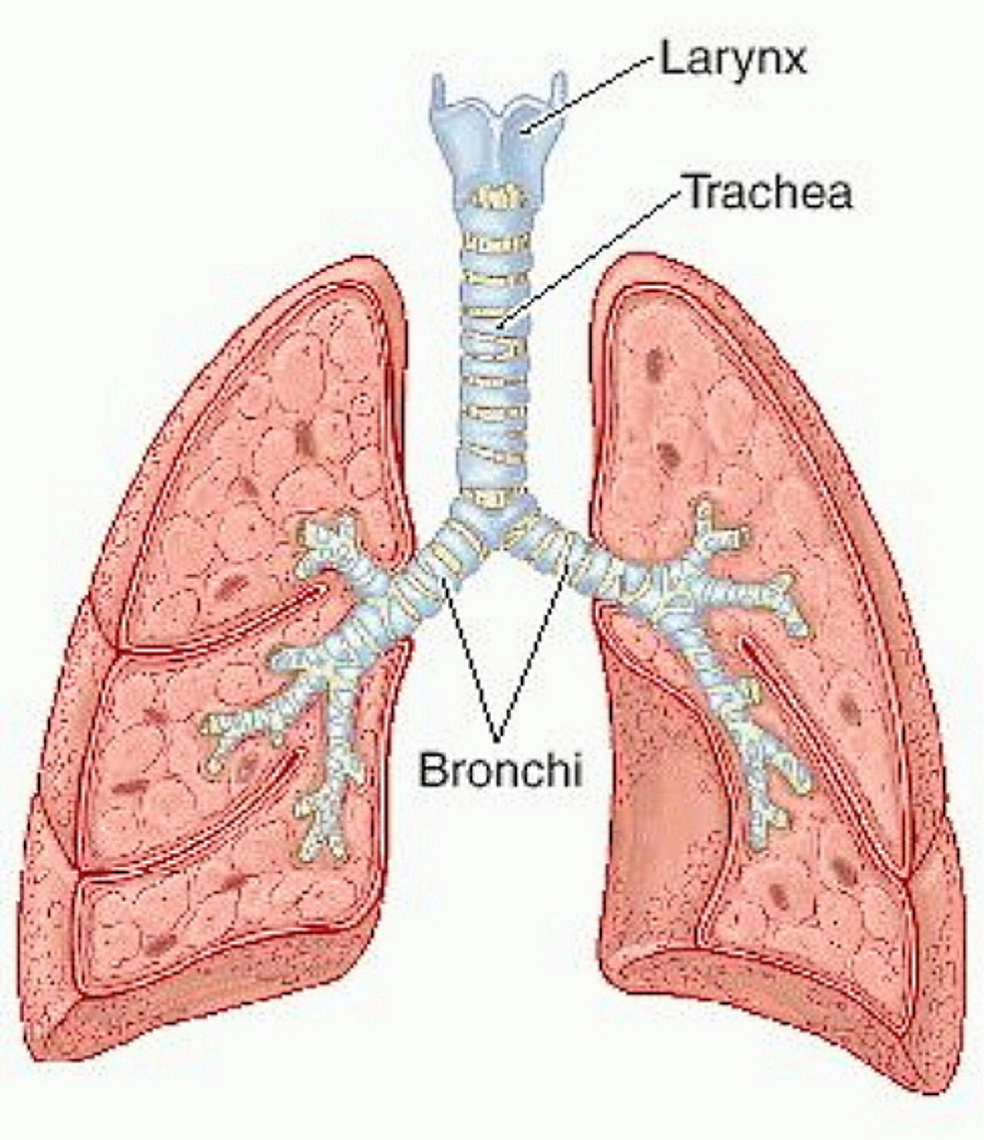
The figure illustrates the subdivision of the bronchi after the carina tracheal. Photographic archive of Bordoni Bruno

The carina divides into the left and right bronchus. The right main bronchus travels caudally for about 2.5 cm (and has an inclination of 25 degrees with respect to the vertical axis), to enter the pulmonary hilum (at the level of T5 [54-55]. The left main bronchus measures about 5 cm (and has an inclination of 45 degrees with respect to the vertical axis and with a slightly posterior orientation), and it enters the pulmonary hilum in a caudal direction (at the level of T6) [54-55]. The pulmonary hilum is found behind the costal cartilages, between the second and fourth ribs, about 2.5 cm from the sternal body (at the level of T4-T6) [54-55]. It is possible to implement palpation of the bronchi before they enter the lung. The carina and the bronchial bifurcation below the sternum are covered by the aortic arch and pulmonary trunk (Figure 2) [57].

**Figure 3:**
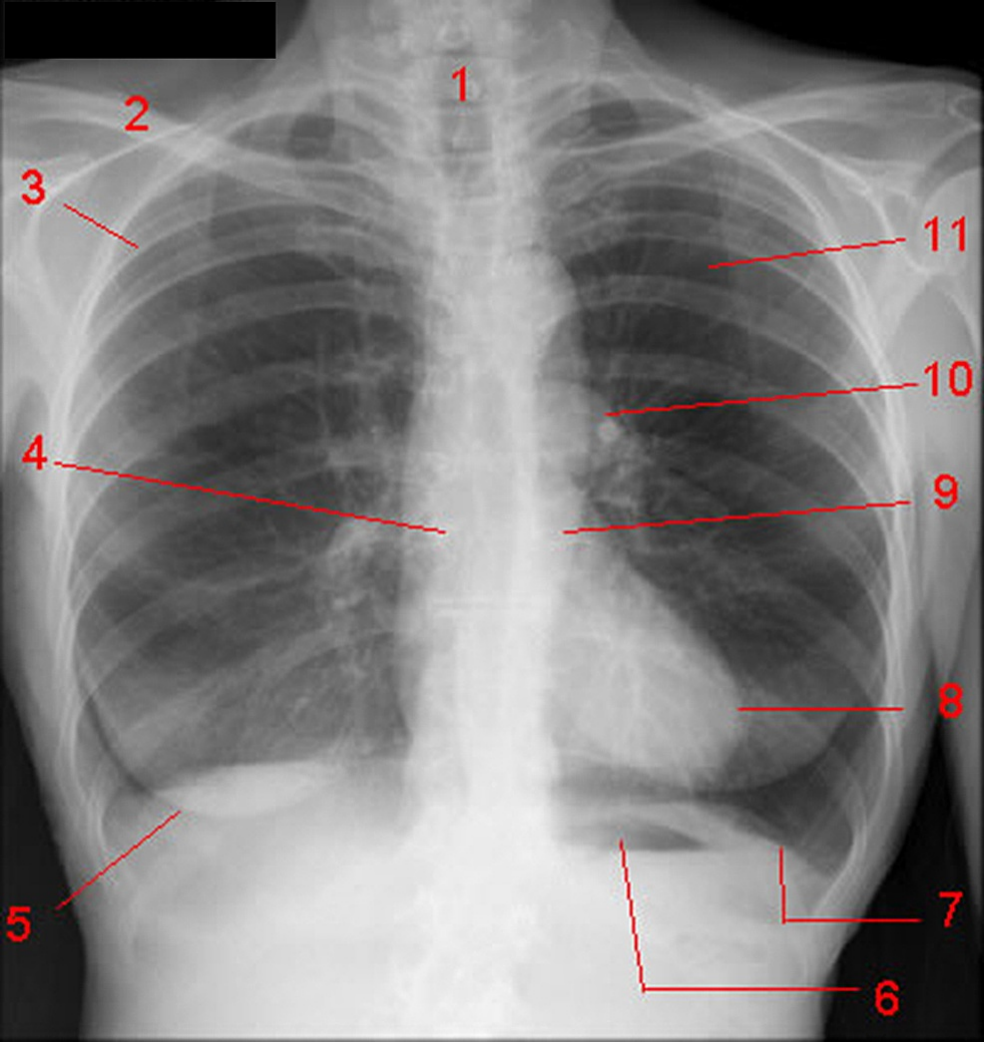
The image shows some anatomical areas that can be deduced from an X-ray of a healthy subject. 1. Trachea. 2. Clavicle. 3. Posterior portion of the fourth rib. 4. Right main bronchus. 5. Lower margin of the right breast. 6. Gastric air bubble. 7. Left diaphragmatic dome. 8. Left ventricle. 9. Descending aorta. 10. Left pulmonary artery. 11. Left lung.

The cervical parietal pleura forms the pleural dome; it rises about 2.5 cm from the line of the medial third of the clavicle until it affects the line of the seventh cervical vertebra [55]. The pleural dome is located behind the sternocleidomastoid muscle and is about 2 cm below the line from the cricoid cartilage [55]. The parietal pleura is covered by the sternum. It descends towards the joint that joins the collarbone to the sternum handlebar, up to the angle of Louis laterally, to continue downwards; once it reaches the fourth costal cartilage, the left pleural line moves laterally to the end of the sixth or seventh costal cartilage due to the presence of the heart [58]. A part of the pleura returns towards the xiphisternal joint while more laterally, it continues in an oblique direction toward the posteriority, up to the twelfth rib [55,58]. Unlike the left pleural line, the right pleural line continues downwards until it reaches the xiphisternal joint laterally [55]. The parietal pleura does not terminate with the diaphragm but continues beyond the presence of the lung, creating the costodiaphragmatic space and costomediastinal space; the costodiaphragmatic recess reaches all the anterior and posterior ribs, covering the diaphragm [55]. The pleural line falls posteriorly downwards, traveling laterally with respect to the vertebral spinous processes and/or laterally to the spinal erector muscles, up to the tenth thoracic vertebra (where the lungs rest on the diaphragm); from here and downwards, the pleurae form the costodiaphragmatic recess [55]. The visceral pleura adhering to the pulmonary parenchyma follow the lines of the parietal pleura, albeit with a millimeter difference; it covers the pulmonary hila and all pulmonary fissures [55]. A fatty layer tends to separate the parietal pleura from the endothoracic fascia, which covers the ribs within the mediastinum (Figure 3) [58].

**Figure 4:**
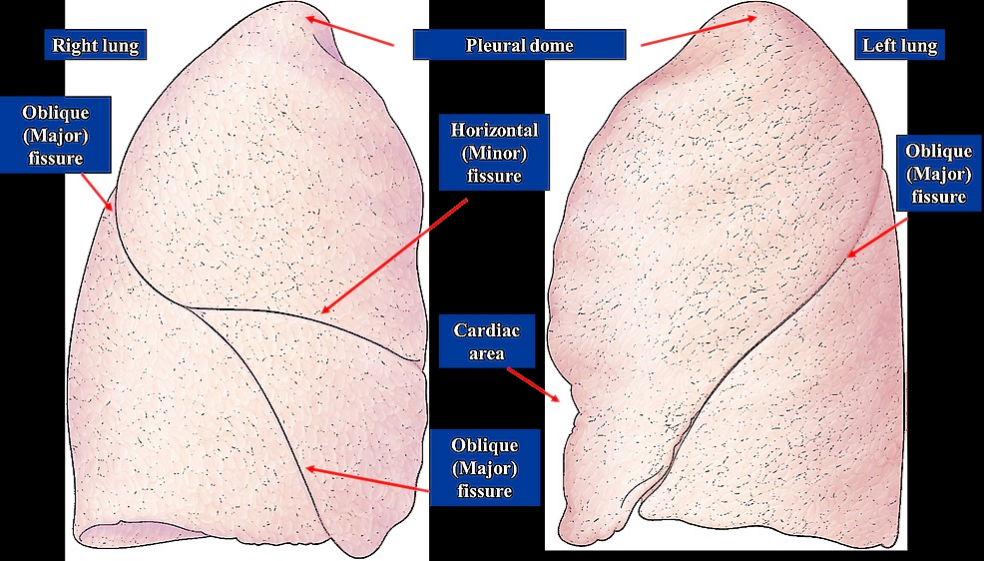
The figure illustrates the different pulmonary fissures of the right lung and the left lung. Photographic archive of Bordoni Bruno

The lobes are covered by the visceral pleura and, through the fissures that separate the lobes, the visceral pleura itself meets itself, allowing the surfaces to slide over one another; this allows independent movement of the single lobes [59]. The left lung is divided into an upper and a lower lobe, which are separated by a major oblique fissure [59]. The left fissure arises posteriorly at the level of the spinous process of the third-fourth thoracic vertebrae, passes through the fifth intercostal space laterally, until arriving anteriorly in the area of the sixth costochondral joint (or above the fifth intercostal space anteriorly) [60]. The right lung has two fissures (and three lobes); namely, the major fissure and the minor or transverse fissure. The right greater fissure arises posteriorly from the area of the fourth spinous process of the thoracic vertebra; basically, it runs along the line of the fifth rib to arrive anteriorly to the sixth costochondral joint [54]. The minor fissure arises from the oblique fissure from the fifth intercostal space laterally, to arrive anteriorly and behind the cartilage of the fourth rib; the minor fissure is not horizontal, but is slightly curved, with cranial convexity (Figure 4) [54,60].

Movement of the respiratory districts

The lower respiratory tract areas are subject to constant movement. From the point of view of OMM, this is a fundamental clue to understanding the health of the underlying tissue. Movement is enabled by the smooth muscle present in the trachea and bronchi, as well as the rich presence of connective tissue in the lungs [36]. Advancing age negatively affects mobility in general, including respiratory structures [61]. The decreased tissue elasticity and increased joint calcification result in reduced compliance of the thoracic cavity and progressive retention of air in the lungs [61]. Senescence itself reduces the contractile capacity of the diaphragm muscle, which negatively affects the compliance of the movement of the respiratory structures [62]. Generally, during inhalation, the trachea undergoes a caudal movement of about 2-3 cm (and back during exhalation), and this movement is caused by negative pulmonary pressures; moreover, it undergoes cranial traction when the craniocervical tract extends by about 2-3 cm, for myofascial relationships [54]. The trachea also undergoes a rotation and lateralization movement when the cervical tract tilts and rotates on the same side; the trachea follows the movement of the neck [63]. The movement of the main bronchi is minimal; the left bronchus (longer than the right main bronchus) moves cranially as the head rotates completely to the right, becoming in line with the trachea [64]. During palpation, clinicians can search for bronchial tissue compliance alongside reduced movement; this area, probably due to its proximity to the pulmonary hilum, is the most fixed during respiratory acts [65]. Pulmonary movements reflect lung function; less movement corresponds to limited respiratory function [66]. With deep breathing, the base of the lung can descend approximately 9-12 cm maximum in healthy subjects [66]. On inhalation, the pulmonary fissures can descend about 2-5 cm; bringing the arms above the shoulders, the medial line of the scapula tends to coincide with the major fissure (right and left) [54-55]. The predominant movement of the lung during respiration is in a cranial-caudal direction, and in millimeters for other lateral-medial movements (3 mm) and in the anteroposterior direction (approximately 2-3 mm) [67-68]. Currently, the literature has reported no data to affirm the existence of clockwise and counterclockwise rotations of the lung lobes. The pleural dome can descend about 6-8 cm during inspiration and has only small displacements for anteroposterior (0.6 cm) and mediolateral movements (0.5 cm) [66].

Palpation of the trachea, main bronchi, and lungs

**Figure 5:**
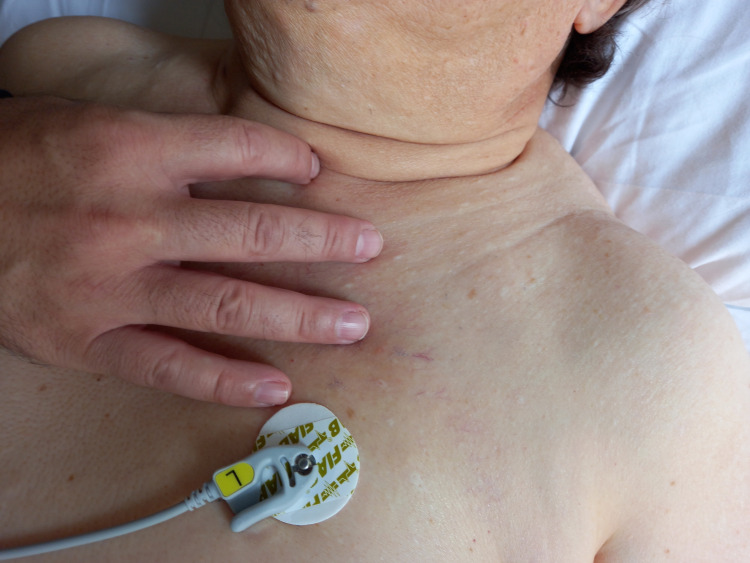
The photo illustrates how to place the fingers on the tracheal rings, to palpate the craniocaudal movement during breaths. The operator stands beside the patient.

We have a duty to update methods according to the ever-changing knowledge. Without first evaluating anatomical areas that are treatment targets, it is not possible to decide which technique to perform. To palpate movement of the trachea, we start from the landmark of the suprasternal notch; from that position, we place the fingers of one hand on the rings of the trachea and wait for the craniocaudal movement (Figure 5). The patient can be asked to extend their head or tilt and rotate their head.

**Figure 6:**
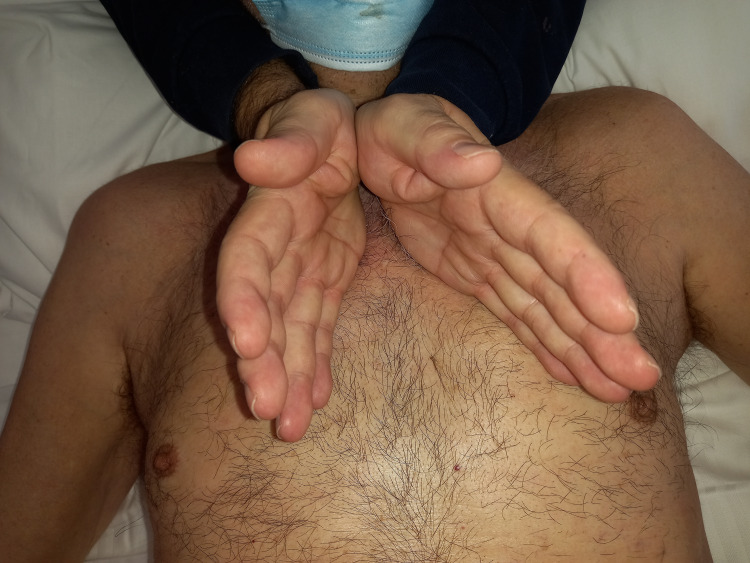
The photo illustrates the operator positioned next to the patient (or behind and to the patient's head) while applying a palpation method for the manual evaluation of the main bronchi, as described in the text.

To palpatorially evaluate the left and right main bronchi, we search for the angle of Louis; the main branches of the bronchi start from here. With the operator at the patient’s side (or behind the patient’s head), the hypothenar eminence is placed on the right bronchial area, on the right sternal body, with an almost vertical orientation. The operator’s left hand rests on the left sternal body and under the angle of Louis, parallel to the right hand but with a more inclined orientation. The operator applies perpendicular stress downwards to test the elasticity of the main bronchi, or the operator may decide to apply a manual tension that reaches the bronchial structure and remain in palpatory listening (Figure 6).

**Figure 7:**
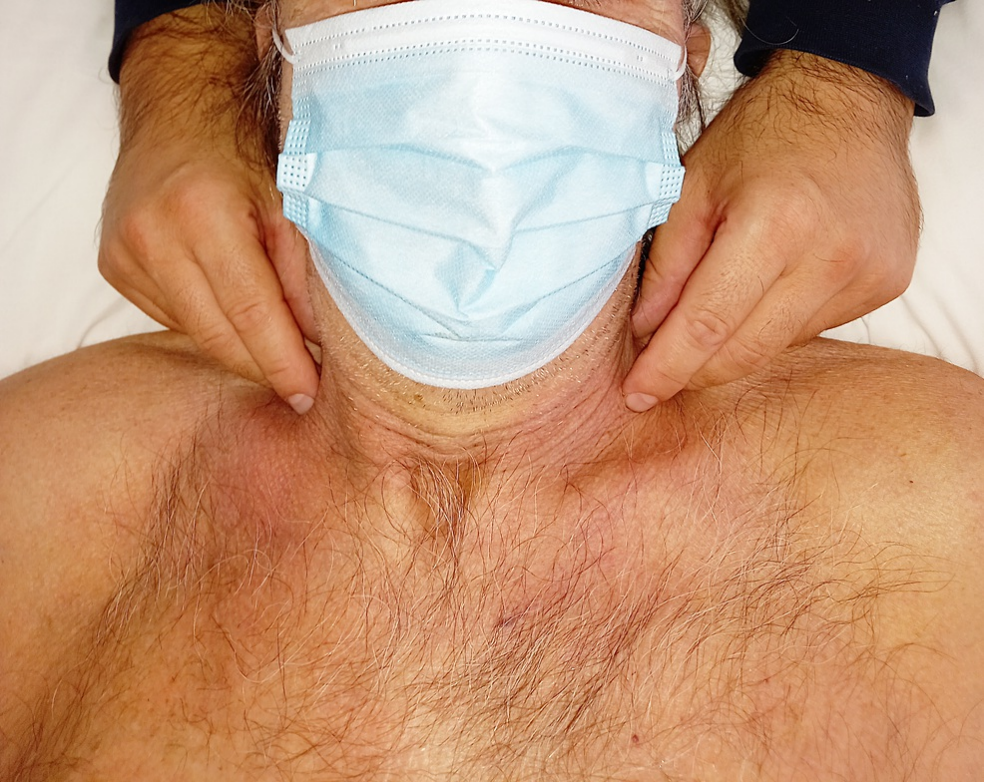
The photo illustrates how the operator must position the fingers of the hands to palpate the movements of the pleural dome, without pressing, but only placing the fingers on the area concomitant with the presence of this anatomical structure.

For the manual evaluation of the pulmonary dome, the operator is behind the patient’s head. Palpation can be done simultaneously on the right and left sides. We locate the trapezius muscle up to the root of the neck (occipital area); the body of the first rib is then identified as the pleural dome enters the internal space created by the first rib. The omohyoid muscle and the stellate ganglion are located above the pleural dome; it is important not to press but use the fingers of one hand to delimit the area of the pulmonary dome as much as possible. The fingers are posterior to, and at the base of, the neck (Figure 7).

**Figure 8:**
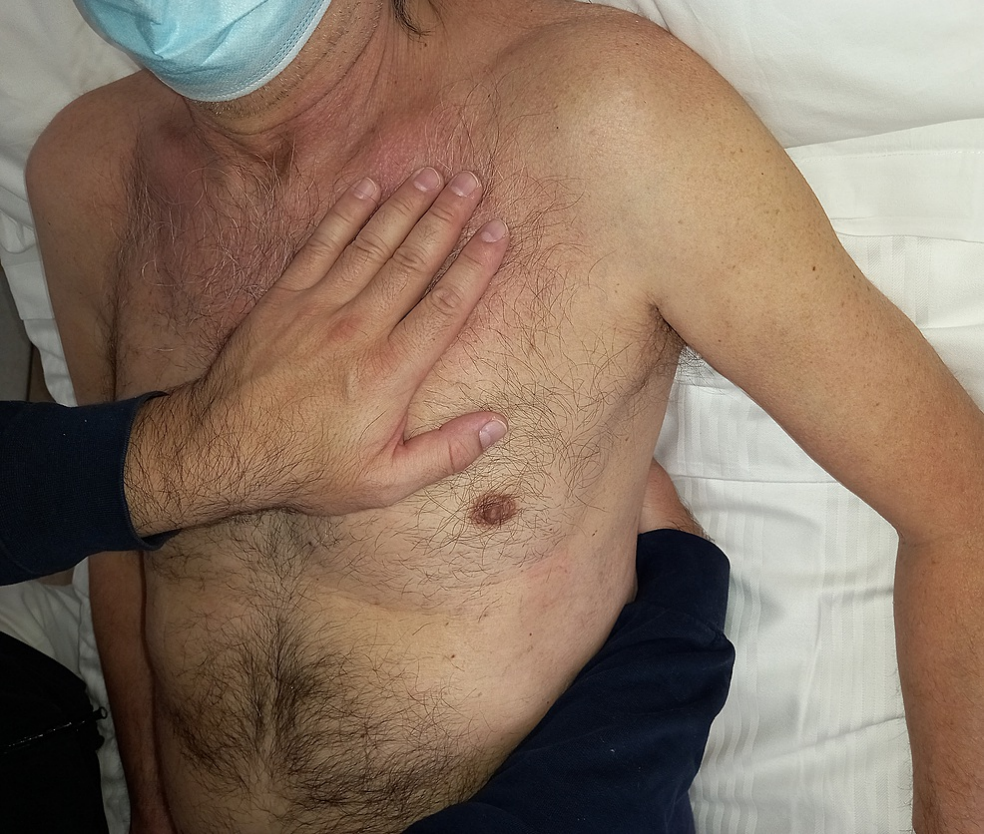
Photo shows palpatory listening to the upper and lower lobes of the left side.

To manually listen to the left lung, the clinician should be on the right side of the patient, who is always in the supine position. The operator’s cranial hand rests on the left costal mediastinal area, without exceeding the “line” of the sixth rib, and in a vertical position; the caudal hand is placed posteriorly, in a horizontal position, until it touches the left paravertebral muscles with the fingers, and without going beyond the tenth dorsal vertebra (Figure 8). The lower lobes are mainly located in the posterior area of the thorax [55]. For palpation of the right lung movement, the clinician should be on the patient’s left side (or on the same side for convenience), with the cranial hand placed vertically on the right anterior rib area, without exceeding the fourth rib. The caudal hand is placed exactly as for manual listening to the lower left lobe but with the hand on the right lobe.

**Figure 9:**
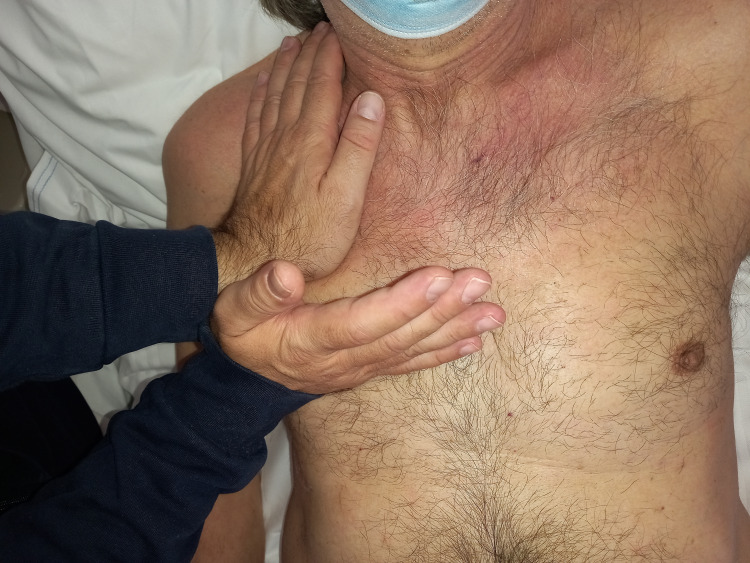
Photo shows how to palpate the movement between the right middle and upper lobe.

To listen to the right middle lobe and compare respiration sound with that of the lower lobe, the cranial hand is placed lower and moved up to involve the ribs anteriorly up to the sixth rib while the caudal hand holds the same position shown in Figure 8. To compare the movement of the right upper lobe with the middle lobe, the clinician holds the cranial hand in the same position for palpatory listening of the right upper lobe, using the caudal hand as vertical support between the fourth and sixth ribs (Figure 9).

Osteopathic physicians can perform palpation of the different tissue textures and related movements; moreover, clinicians can direct palpatory attention towards the dysfunction. With osteopathic evaluation, clinicians can decide how to intervene to help restore homeostasis, which must be as healthy as possible [69]. For the evaluation of pulmonary recesses, indirect listening is possible through the movement of the diaphragm muscle and the palpation process, as reported in the literature [70]. The ergonomic position taken by the clinician may change, as the osteopath must adapt to the patient’s ability to remain in the required posture. Furthermore, the patient can be placed in the lateral decubitus position for the same palpatory listening.

## Conclusions

The article reviews the topographical anatomy of the trachea, main bronchi, and lungs. We reviewed the neurological anatomy of the lower airways and, briefly, the importance of the passage of air and fluids with respect to responses to respiratory form and function. This is the first article to illustrate how to perform palpation on these anatomical areas, drawing upon the most up-to-date anatomical knowledge. Before deciding which manual strategy to apply to a patient, osteopathic physicians must perform palpation of dysfunctional areas; in this way, clinicians can also re-evaluate the same areas of a given patient after the treatment.
